# Role of Insect Gut Microbiota in Pesticide Degradation: A Review

**DOI:** 10.3389/fmicb.2022.870462

**Published:** 2022-05-03

**Authors:** Junaid Ali Siddiqui, Muhammad Musa Khan, Bamisope Steve Bamisile, Muhammad Hafeez, Muhammad Qasim, Muhammad Tariq Rasheed, Muhammad Atif Rasheed, Sajjad Ahmad, Muhammad Ibrahim Shahid, Yijuan Xu

**Affiliations:** ^1^Department of Entomology, South China Agricultural University, Guangzhou, China; ^2^State Key Laboratory of Rice Biology, Institute of Insect Sciences, Zhejiang University, Hangzhou, China; ^3^Department of Agriculture and Forestry, Kohsar University Murree, Punjab, Pakistan; ^4^Department of Life Sciences, Khwaja Fareed University of Engineering and Information Technology, Rahim Yar Khan, Pakistan; ^5^Department of Entomology, Pir Mehr Ali Shah Arid Agriculture University, Rawalpindi, Pakistan; ^6^Key Laboratory of Integrated Pest Management of Crop in South China, Ministry of Agriculture and Rural Affairs, Guangzhou, China; ^7^Key Laboratory of Natural Pesticide and Chemical Biology, Ministry of Education, South China Agricultural University, Guangzhou, China; ^8^Department of Entomology, University of Faisalabad, Faisalabad, Pakistan

**Keywords:** toxicology, microbial detoxification, insecticide degradation, resistant species, symbiotic bacteria

## Abstract

Insect pests cause significant agricultural and economic losses to crops worldwide due to their destructive activities. Pesticides are designed to be poisonous and are intentionally released into the environment to combat the menace caused by these noxious pests. To survive, these insects can resist toxic substances introduced by humans in the form of pesticides. According to recent findings, microbes that live in insect as symbionts have recently been found to protect their hosts against toxins. Symbioses that have been formed are between the pests and various microbes, a defensive mechanism against pathogens and pesticides. Insects’ guts provide unique conditions for microbial colonization, and resident bacteria can deliver numerous benefits to their hosts. Insects vary significantly in their reliance on gut microbes for basic functions. Insect digestive tracts are very different in shape and chemical properties, which have a big impact on the structure and composition of the microbial community. Insect gut microbiota has been found to contribute to feeding, parasite and pathogen protection, immune response modulation, and pesticide breakdown. The current review will examine the roles of gut microbiota in pesticide detoxification and the mechanisms behind the development of resistance in insects to various pesticides. To better understand the detoxifying microbiota in agriculturally significant pest insects, we provided comprehensive information regarding the role of gut microbiota in the detoxification of pesticides.

## Introduction

Insects are the world’s most diverse and abundant animals in terms of species diversity and body mass in all ecological habitats (Nagarajan et al., 2022). Their numerous interactions with beneficial microbes are essential for survival and diversity. Microbes that are living in the guts of insects play a vital role in the biology and behavior of their hosts, including assisting in the digestion of recalcitrant food components, upgrading nutrient-poor diets, modulating the immune response, and protecting from predators, parasites, pathogens, and disease vectors. Other functions include facilitating plant specialization, governing mating preference and reproductive systems, and contributing to inter- and intraspecific communication (Sharon et al., 2010; Engel et al., 2012; Tokuda et al., 2018; Xia et al., 2018).

Many studies describing symbiotic connections between microbes and insects have been published (Funaro et al., 2011; Dang et al., 2017; Nicoletti and Becchimanzi, 2022). Most insects are thought to be in symbiotic partnerships with microbes, with estimates ranging from 15 to 20% of the total (Zhou et al., 2021). The role of microorganisms, particularly gut microbes, in insect function is important from various viewpoints, including agriculture, ecology, and medicine. Few insects are good laboratory models for studying microbe populations and their associations with hosts, especially immunology and metabolic associations (Hamilton and Perlman, 2013). Entomological studies of parasitic and mutualistic connections have focused on social insects like ants, which have evolved diverse interactions with other species at various levels, including individual and community interactions. These interactions can occur between bacteria and different insects and plants (Moreau, 2020).

Symbiotic bacteria can affect the efficacy of disease vectors or their developmental time, making them possible targets for disease control (Chouaia et al., 2012; Ricci et al., 2012). Microorganisms allied with pollinators and herbivores, and insects that feed on them are likely to impact the agricultural crops’ health substantially. Insects and their gut microbial populations play vital roles in the nitrogen cycle and the decomposition of plant material in natural and human-impacted ecosystems (Fox-Dobbs et al., 2010; Engel and Moran, 2013). A symbiotic relationship with very adaptable bacteria may have opened new ecological niches and unbalanced food sources like plant sap or blood (Sudakaran et al., 2017). Mutualism between insects and microbes is unquestionably one of the primary drivers of insect evolution. It is one of the most important factors contributing to the remarkable success of this gigantic group of animals. Mutualism is described as an interaction between various species mutually advantageous to both parties (Armitage et al., 2022). Several fitness traits of insects are heavily influenced by associated microbiota (de Almeida et al., 2017). The association of insects with microbiota is very important for the evolution of ecological features and feeding habits in which insects exchange nutrients or specific functions, such as protection from adversaries or transit between parties (Kikuchi et al., 2012; Suárez-Moo et al., 2020). Symbiotic-associated bacteria allow insects to feed on hard-to-digest and nutritionally poor diets (Salem and Kaltenpoth, 2022). However, insects may be associated with various microbes that also play an important role in degrading pesticides.

Pesticides may have unintended harmful impacts on humans, non-target creatures, and the environment (surface, soils, and groundwater), as the products are designed to be poisonous and are intentionally discharged into the environment (Kamal et al., 2020). Pesticide hazard is a function of the pesticide’s (eco) toxicological qualities and the pesticide’s ability to harm humans, flora, and animals (Müller et al., 2014). In modern farming systems, pesticides have become an important part of the process. As a result of persistent pests’ resurgence, the overreliance on pesticides for pest control may not end soon. Consequently, various biological and ecological factors mediate several available reports on insect pests’ resistance against different pesticides (Table 1). As a result of the overdependence on synthetic pesticides, numerous concerns have been raised in lieu of their side effects, such as the development of resistance in the target insects, the pollution of the environment, and the effects on human health (Du et al., 2020). It has also been suggested that pesticide resistance may be influenced by gut microbiota, which adds another degree of complexity to the processes of resistance (Gressel, 2018). Bacteria have been demonstrated to directly break down organic pesticides such as chlorpyrifos, dimethoate, and ethoprophos (Nayak et al., 2018; Chen et al., 2020; Gunstone et al., 2021). Furthermore, agricultural pests regularly acquire these bacteria after ingesting them from various sources, including food and the environment (Kikuchi et al., 2012). The gut microbiome may also potentially aid in detoxification by modulating the immune system of the host (Xia et al., 2018). Gut bacteria that produce nutrients and other beneficial chemicals may help the host develop better and increased tolerance to food poisons, although direct experimental data remains sparse (Kohl and Dearing, 2016; Mason et al., 2019).

**TABLE 1:** Some of the common pesticides that have been used against various resistant insect pests.

Pesticides	Common name of the targeted insect pests	Scientific name	References
Abamectin	American serpentine leafminer	*Liriomyza trifolii*	Ferguson, 2004
	Beet armyworm	*Spodoptera exigua*	Ishtiaq et al., 2012
	Cotton leafworm	*Spodoptera litura*	Ahmad et al., 2008
Acetamiprid	Melon and cotton aphid	*Aphis gossypii*	Bass et al., 2015
	Tobacco whitefly	*Bemisia tabaci*	Basit et al., 2013
	Asian citrus psyllid	*Diaphorina citri*	Naeem et al., 2016
	Colorado potato beetle	*Leptinotarsa decemlineata*	Bass et al., 2015
	Rice planthoppers	*Sogatella furcifera*	Zhang et al., 2017
	Codling moth	*Cydia pomonella*	Bass et al., 2015
	Cotton leafhopper	*Amrasca biguttula biguttula*	Chaudhari et al., 2015
	Western flower thrips	*Frankliniella occidentalis*	Bass et al., 2015
Azadirachtin	Tobacco whitefly	*Bemisia tabaci*	Dângelo et al., 2018
Benfuracarb	Melon and cotton aphid	*Aphis gossypii*	Koo et al., 2014; Bass et al., 2015
Bifenthrin	Melon and cotton aphid	*Aphis gossypii*	Koo et al., 2014; Bass et al., 2015
Buprofezin	Tobacco whitefly	*Bemisia tabaci*	Basit et al., 2013
	Rice planthoppers	*Sogatella furcifera*	Zhang et al., 2014
	The brown planthopper	*Nilaparvata lugens*	Wu S. F. et al., 2018
	Rice planthoppers	*Sogatella furcifera*	Jin et al., 2017
Carbamate	Cotton leafworm	*Spodoptera litura*	Saleem et al., 2008
Chlorantraniliprole	Beet armyworm	*Spodoptera exigua*	Lai and Su, 2011
	Tomato leafminer	*Tuta absoluta*	Roditakis et al., 2018
Chlorpyrifos	Rice planthoppers	*Sogatella furcifera*	He et al., 2015; Jin et al., 2017
	Beet armyworm	*Spodoptera exigua*	Ishtiaq et al., 2012
Chlorpyriphos	Asian citrus psyllid	*Diaphorina citri*	Naeem et al., 2016; Chen et al., 2021
Clothianidin	Melon and cotton aphid	*Aphis gossypii*	Koo et al., 2014; Bass et al., 2015
	Colorado potato beetle	*Leptinotarsa decemlineata*	Bass et al., 2015
	Green peach aphid	*Myzus persicae*	Bass et al., 2015
	Rice planthoppers	*Sogatella furcifera*	Zhang et al., 2017
	The brown planthopper	*Nilaparvata lugens*	Khan et al., 2020
Cypermethrin	Beet armyworm	*Spodoptera exigua*	Ishtiaq et al., 2012; Hafeez et al., 2020
Cyromazine	American serpentine leafminer	*Liriomyza trifolii*	Ferguson, 2004
Deltamethrin	Melon and cotton aphid	*Aphis gossypii*	Koo et al., 2014; Bass et al., 2015
	Tobacco whitefly	*Bemisia tabaci*	Longhurst et al., 2013
	Beet armyworm	*Spodoptera exigua*	Ishtiaq et al., 2012; Hafeez et al., 2019
	Red flour beetle	*Tribolium castaneum*	Zhu et al., 2016
Diamide	Diamondback moth	*Plutella xylostella*	Steinbach et al., 2015
	Tomato leafminer	*Tuta absoluta*	Roditakis et al., 2017
Diflubenuron	Cotton leafworm	*Spodoptera litura*	Ahmad et al., 2008
Dinotefuran	Melon and cotton aphid	*Aphis gossypii*	Koo et al., 2014; Bass et al., 2015
	Colorado potato beetle	*Leptinotarsa decemlineata*	Bass et al., 2015
	Rice planthoppers	*Sogatella furcifera*	Zhang et al., 2017
Emamectin benzoate	Housefly	*Musca domestica*	Khan et al., 2016
	Diamondback moth	*Plutella xylostella*	Patil et al., 2011
	Beet armyworm	*Spodoptera exigua*	Ishtiaq et al., 2012
	Tomato leafminer	*Tuta absoluta*	Roditakis et al., 2018
Esfenvalerate	Melon and cotton aphid	*Aphis gossypii*	Koo et al., 2014; Bass et al., 2015
Ethiprole	The brown planthopper	*Nilaparvata lugens*	Garrood et al., 2016
Fenpropathrin	Asian citrus psyllid	*Diaphorina citri*	Tiwari et al., 2011
Fenvalerate	Beet armyworm	*Spodoptera exigua*	Musa Khan et al., 2021
Fipronil	Diamondback moth	*Plutella xylostella*	Wang et al., 2016a
	Cotton leafworm	*Spodoptera litura*	Ahmad et al., 2008
	Rice planthoppers	*Sogatella furcifera*	Tang et al., 2010; Jin et al., 2017
Flonicamid	Melon and cotton aphid	*Aphis gossypii*	Koo et al., 2014; Bass et al., 2015
Imidacloprid	Melon and cotton aphid	*Aphis gossypii*	Koo et al., 2014; Bass et al., 2015; Kim et al., 2015
Imidacloprid	Asian citrus psyllid	*Diaphorina citri*	Bass et al., 2015
	Small brown planthopper	*Laodelphax striatellus*	Bass et al., 2015
	Housefly	*Musca domestica*	Bass et al., 2015
	Green peach aphid	*Myzus persicae*	Bass et al., 2015
	The brown planthopper	*Nilaparvata lugens*	Bass et al., 2015; Garrood et al., 2016; Wu S. F. et al., 2018
	Avocado thrips	*Scirtothrips perseae*	Byrne et al., 2005
	Rice planthoppers	*Sogatella furcifera*	Bass et al., 2015
	Cotton leafhopper	*Amrasca biguttula biguttula*	Chaudhari et al., 2015
	Tobacco whitefly	*Bemisia tabaci*	Longhurst et al., 2013
	Asian citrus psyllid	*Diaphorina citri*	Tiwari et al., 2011; Naeem et al., 2016
	Western flower thrips	*Frankliniella occidentalis*	Bass et al., 2015
	Colorado potato beetle	*Leptinotarsa decemlineata*	Bass et al., 2015
	Rice planthoppers	*Sogatella furcifera*	Jin et al., 2017
	Greenhouse whitefly	*Trialeurodes vaporariorum*	Bass et al., 2015
	Tobacco whitefly	*Bemisia tabaci*	Hamada et al., 2019
	Chinese chive maggot	*Bradysia odoriphaga*	Chen et al., 2019
	Colorado potato beetle	*Leptinotarsa decemlineata*	Kalsi and Palli, 2017
	Asian citrus psyllid	*Diaphorina citri*	Kalsi and Palli, 2017
	The brown planthopper	*Nilaparvata lugens*	Hamada et al., 2020; Khan et al., 2020
	Grain aphid	*Sitobion avenae Fabricius*	Zhang et al., 2020a
	The western flower thrips	*Frankliniella occidentalis*	Wan et al., 2018
Imidaclothiz	Western flower thrips	*Frankliniella occidentalis*	Bass et al., 2015
Indoxacarb	Beet armyworm	*Spodoptera exigua*	Ishtiaq et al., 2012
	Cotton leafworm	*Spodoptera litura*	Ahmad et al., 2008
	Tomato leafminer	*Tuta absoluta*	Roditakis et al., 2018
	Red imported fire ant	*Solenopsis invicta*	Siddiqui et al., 2022
Lambda-cyhalothrin	Tobacco whitefly	*Bemisia tabaci*	Dângelo et al., 2018
	Brown stink bug	*Euschistus heros*	Hegeto et al., 2015
	Fall armyworm	*Spodoptera frugiperda*	Hafeez et al., 2021
Lufenuron	Cotton leafworm	*Spodoptera litura*	Ahmad et al., 2008
	Beet armyworm	*Spodoptera exigua*	Ishtiaq et al., 2012
Malathion	Asian citrus psyllid	*Diaphorina citri*	Tiwari et al., 2011
Methamidophos	Brown stink bug	*Euschistus heros*	Sosa-Gómez and da Silva, 2010
Methoxyfenozide	Housefly	*Musca domestica*	Shah et al., 2017
	Beet armyworm	*Spodoptera exigua*	Ishtiaq et al., 2012
	Cotton leafworm	*Spodoptera litura*	Ahmad et al., 2008
Neonicotinoids	Green peach aphid	*Myzus persicae*	Panini et al., 2014
Nitenpyram	Asian citrus psyllid	*Diaphorina citri*	Naeem et al., 2016
	Rice planthoppers	*Sogatella furcifera*	Zhang et al., 2017
	Tobacco whitefly	*Bemisia tabaci*	Basit et al., 2013
	The brown planthopper	*Nilaparvata lugens*	Khan et al., 2020
Organochlorinc	Cotton leafworm	*Spodoptera litura*	Saleem et al., 2008
Organophosphate	Cotton leafworm	*Spodoptera litura*	Saleem et al., 2008
Organophosphates	Currant–lettuce aphid	*Nasonovia ribisnigri*	Barber et al., 1999
	Beet armyworm	*Spodoptera exigua*	Ishtiaq et al., 2012
	Greenhouse whitefly	*Trialeurodes vaporariorum*	Bass et al., 2015
Organophosphorus	Colorado potato beetle	*Leptinotarsa decemlineata*	Malekmohammadi and Galehdari, 2016
	Onion thrips	*Thrips tabaci*	Nazemi et al., 2016
Phenylpyrazole	The brown planthopper	*Nilaparvata lugens*	Garrood et al., 2017
Pirimicarb	Currant–lettuce aphid	*Nasonovia ribisnigri*	Barber et al., 1999
Profenofos	Beet armyworm	*Spodoptera exigua*	Ishtiaq et al., 2012
	Tobacco whitefly	*Bemisia tabaci*	Longhurst et al., 2013
Pymetrozine	Greenhouse whitefly	*Trialeurodes vaporariorum*	Bass et al., 2015
	Rice planthoppers	*Sogatella furcifera*	Jin et al., 2017
Pyrethroids	German cockroach	*Blattella germanica*	Wei et al., 2001
	Pollen beetle	*Meligethes aeneus*	Zimmer and Nauen, 2011
	The brown planthopper	*Nilaparvata lugens*	Sun et al., 2017
	Diamondback moth	*Plutella xylostella*	Sonoda et al., 2012
	Cabbage stem flea beetle	*Psylliodes chrysocephala*	Zimmer et al., 2014; Højland et al., 2015
	Grain aphid	*Sitobion avenae*	Foster et al., 2014
	Cotton leafworm	*Spodoptera litura*	Saleem et al., 2008
	Onion thrips	*Thrips tabaci*	Toda and Morishita, 2009; Nazemi et al., 2016
	Green peach aphid	*Myzus persicae*	Panini et al., 2014
	Greenhouse whitefly	*Trialeurodes vaporariorum*	Bass et al., 2015
	Currant–lettuce aphid	*Nasonovia ribisnigri*	Barber et al., 1999
	Beet armyworm	*Spodoptera exigua*	Ishtiaq et al., 2012
Spinetoram	Western flower thrips	*Frankliniella occidentalis*	Wang et al., 2016b
Spinosad	Oriental fruit fly	*Bactrocera dorsalis*	Sparks et al., 2012
	Olive fruit fly	*Bactrocera oleae*	Sparks et al., 2012
	Braconid wasp	*Cotesia plutellae*	Sparks et al., 2012
	Fruit fly	*Drosophila melanogaster*	Sparks et al., 2012
	Cotton bollworm	*Helicoverpa armigeria*	Sparks et al., 2012
	Tobacco budworm	*Heliothis virescens*	Sparks et al., 2012
	Oblique-banded leafroller	*Lepidoptera Choristoneura rosaceana*	Sparks et al., 2012
	American serpentine leafminer	*Liriomyza trifolii*	Sparks et al., 2012
	American serpentine leafminer	*Liriomyza trifolii*	Ferguson, 2004
	Housefly	*Musca domestica*	Sparks et al., 2012
	Diamondback moth	*Plutella xylostella*	Sparks et al., 2012
	Beet armyworm	*Spodoptera exigua*	Ishtiaq et al., 2012; Sparks et al., 2012
	Cotton leafworm	*Spodoptera litura*	Ahmad et al., 2008
	The western flower thrips	*Frankliniella occidentalis*	Sparks et al., 2012
	Tomato leafminer	*Tuta absoluta*	Silva et al., 2016
	Western flower thrips	*Frankliniella occidentalis*	Wang et al., 2016b
Spiromesifen	Tobacco whitefly	*Bemisia tabaci*	Dângelo et al., 2018
Sulfoxaflor	Melon and cotton aphid	*Aphis gossypii*	Koo et al., 2014; Bass et al., 2015
Thiacloprid	Melon and cotton aphid	*Aphis gossypii*	Koo et al., 2014; Bass et al., 2015
	Tobacco whitefly	*Bemisia tabaci*	Basit et al., 2013
	Codling moth	*Cydia pomonella*	Bass et al., 2015; İşci and Ay, 2017
	Colorado potato beetle	*Leptinotarsa decemlineata*	Bass et al., 2015
	Pollen beetle	*Meligethes aeneus*	Zimmer and Nauen, 2011
Thiamethoxam	Cotton leafhopper	*Amrasca biguttula biguttula*	Chaudhari et al., 2015
	Melon and cotton aphid	*Aphis gossypii*	Koo et al., 2014; Bass et al., 2015
	Asian citrus psyllid	*Diaphorina citri*	Bass et al., 2015
	Brown stink bug	*Euschistus heros*	Hegeto et al., 2015
	Housefly	*Musca domestica*	Bass et al., 2015
	Asian citrus psyllid	*Diaphorina citri*	Tiwari et al., 2011; Naeem et al., 2016
	Western flower thrips	*Frankliniella occidentalis*	Bass et al., 2015
	The brown planthopper	*Nilaparvata lugens*	Wu S. F. et al., 2018; Khan et al., 2020
	Rice planthoppers	*Sogatella furcifera*	Jin et al., 2017

Increasing apprehensions about the dramatic upsurge in pesticide resistance in pests have prompted researchers to better understand the mechanisms through which insect gut microbiome may confer resistance. Insect gut microbial populations have been studied for their potential role in pesticide resistance—for example, in *Riptortus pedestris*, *Burkholderia* symbionts have been demonstrated to promote pesticide resistance, and fenitrothion-degrading *Burkholderia* strains can also be shifted horizontally to other insects (Kikuchi and Yumoto, 2013). Similarly, Cheng et al. (2017) found that trichlorfon-degrading *Citrobacter* sp. (CF-BD) isolated from the gut of *Bactrocera dorsalis* increased pesticide resistance in the cockroach gut. In addition, many non-septate fungi and bacteria, assumed to be mutualistic, were found in the small intestines of workers of cephalotinid ants. These bacteria live as a moderately dense flora that contains a diverse range of bacterial species, including gram-positive and gram-negative coccobacilli and anaerobes similar to *Bacterioides* and *Clostridia* species (Donelli et al., 2012).

We already know that the environment in the insect gut regulates or even determines the shape of the community microbiota diversity and its metabolic activities, which might cause physical consequences for insects (Tang et al., 2012; Xia et al., 2018). Variations in environmental situations have been shown to affect the microbiota interrelationships among insects and their microbiota and related gene expression (Possemiers et al., 2011; Stencel and Wloch-Salamon, 2018). Recently, emerging research have suggested associations between insect gut microbiome and pesticide resistance. Several studies ranging from community diversity surveys to molecular analyses have focused on the gut bacteria’s interactions with the host immune systems (Kikuchi et al., 2012; Engel and Moran, 2013; Xia et al., 2013; Chmiel et al., 2019).

However, despite compelling reasons to further understand the roles played by insect gut microorganisms and a recent increase in research on microbes that live in insect guts, there has been little progress in expanding the available knowledge on the role of insect gut microbiota in the degradation of pesticides. Currently, pest resistance issues need to be addressed, so the current review will explore the functions and mechanism of pesticide resistance aided by gut microbiota and elaborate their role in pesticide degradation.

## Insect Gut Structure and Functions

**FIGURE 1:**
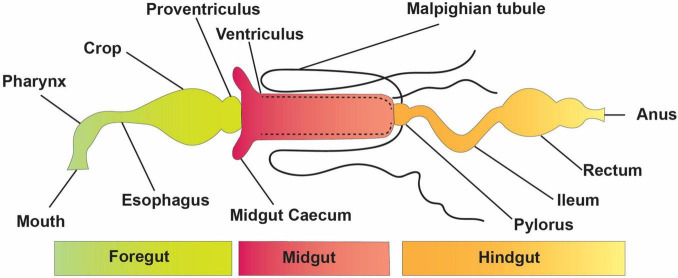
Basic structure and divisions of the insect digestive system.

The elementary structure of the intestinal system is alike among insects, even though they have a variety of alterations connected with adaptation to diverse feeding styles and environmental conditions (Figure 1). The digestive tract is divided into three basic regions: the foregut, the midgut, and the hindgut (Simpson, 2013). The foregut and hindgut originate from the embryonic epithelium and are protected from pathogens by an exoskeleton of chitin and integument glycoproteins. This exoskeleton is shed at each ecdysis, separating the gastrointestinal lumen from the epithelia. When divided into functionally different subgroups, the foregut is frequently distinguished by another diverticula or crop for impermanent food storage (Linser and Dinglasan, 2014). The hindgut includes distinct portions like fermentation compartments and a distinct rectum for retaining feces during earlier evacuation, among other things. In many insects, the midgut is the main location of absorption and digestion. It lacks an exoskeletal lining and develops from endodermal cells rather than the rest of the body (Engel and Moran, 2013). A protective envelope known as the peritrophic matrix (or peritrophic membrane) is released by the midgut epithelial cells of many insects. This envelope, constantly being renewed as lost, is essential for the insect’s survival. The midgut has two parts: the endo- and ectoperitrophic space. Microorganisms are generally kept in the endo-peritrophic area, which prevents them from coming into direct contact with the epithelium. Peritrophic matrixes are classified into two discrete categories, namely, type I and type II. Type I refers to the whole midgut and is occasionally active when particular foods are consumed, whereas type II is in the remote location of the anterior mid-gut (Engel and Moran, 2013).

The peritrophic matrix shields the epithelium against mechanical injury by food elements, toxins in food, invasive microbes, and absorbed food and digestive enzymes (Kuraishi et al., 2013; Dastranj et al., 2016). In other circumstances, the peritrophic medium wraps around the undigested food mass as it passes along the digestive tract. Tiny pores in the peritrophic matrix prevent most microbes from passing through while allowing enzymes and small molecules from digested food to get through (Terra and Ferreira, 2012; Engel and Moran, 2013). Several insect species, including most sap-feeding species (Hemiptera), various other species of family Formicidae, and order Coleoptera (Nardi and Bee, 2012), that rely solely on cell sap or honeydew do not form a peritrophic matrix (Engel and Moran, 2013).

The Malpighian tubules of insects are excretory structures that extend from the anterior hindgut into the body void and ingest wastes, such as uric acid supplied to the hindgut (Figure 1). As a result, the hindgut of insects comprises a distinct nutritional environment which is well documented for water resorption (Simpson, 2013); the hindgut might function as a location of nutrient assimilation, as verified for different insect pests, including termites (Ayitso and Onyango, 2016), crickets (Smith et al., 2017), cockroaches (Tinker and Ottesen, 2016), and heteropteran (Gutiérrez-Cabrera et al., 2016)—for instance, intercellular passages in the hindgut membrane of several cockroaches permit nutrients, such as amino acids and fatty acids made *via* the biota in the hindgut, to flow from the hindgut lumen to the insect hemolymph (O’Donnell and Donini, 2017). The basic form of an insect gut has undergone numerous alterations due to adaptations to specialized niches and eating patterns.

## Insect Gut Microbiome Composition

A wide range of parameters can influence gut microbiota composition, including insect growth, biochemical changes in different intestinal areas, and the insect’s ability to obtain available resources (Bruno et al., 2019b). The hindgut of insects, which serves as an extension of the body cavity, is one of these structures that collect dietary waste. Therefore, it provides a great food environment to the gut microbiota, encouraging their proliferation and diversification (Engel and Moran, 2013; Bruno et al., 2019a).

The insect gut microbiome includes protozoa, fungus, archaea, and bacteria. Protists occupy almost 90% of the hindgut of subterranean termites—for example, lower and higher termites’ guts include bacteria and archaea (Hongoh, 2010). Scientists revealed that the digestive regions of adult workers of honeybee (*Apis mellifera*) are dominated by a diverse group of nine bacterial species (five of which are *Snodgrassella alvi* and *Gilliamella apicola*, two species of *Lactobacillus*, and a species of *Bifidobacterium*) (Douglas, 2018). Additionally, the gut microbiota is rarely directly touched with intestinal epithelial cells due to their unique placement. Most of the time, bacteria that live in the gut are found in the lumen of the endoperitrophic space, a chitinous barrier that lines the middle of the gut (Erlandson et al., 2019). Yun et al. (2014) have comprehensively categorized and thoroughly defined the insect-associated gut bacteria of 305 samples belonging to 218 species in 21 taxonomic orders. The results indicated that Proteobacteria and Firmicutes were found to make up 62.1 and 20.7% of the total reads in the insect gut microbiota, respectively. Moreover, *Wolbachia* made up 14.1% of the total reads.

## Interaction of Insect and Their Related Microbiota

**FIGURE 2:**
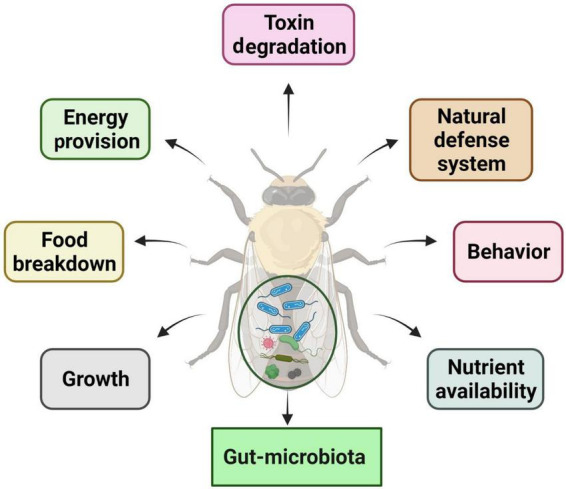
Functions and impacts of gut microbiota on insect biology and physiology.

Insect–microbiota interactions are quite diverse. Insects rely on symbiotic bacteria for a variety of essential activities. Symbiotic bacteria can be critical for host survival and growth (Consortium, 2012; Douglas, 2015; Berasategui et al., 2016). They can help break down food, provide energy, make vitamins, and even help shape the body’s natural defenses (Cheng et al., 2019; Figure 2). Microbial symbionts have been proven to have many consequences on insect health and behavior (Sampson and Mazmanian, 2015). Certain insects have specialized organs that can only house a few symbiont species, while others have a far more diverse and variable flora in their guts and other internal organs. Numerous associations are developed with a sole or a few species of microbiota. They might require establishing specialized insect organs and cells (i.e., subsequent midgut crypts, mycangia, and microbiome) to house definite obligate symbionts (Zaidman-Rémy et al., 2018; Kuechler et al., 2019; Maire et al., 2019; Trappeniers et al., 2019). In these partnerships, the genetic integral of biochemical processes essential for the persistence of both interrelating groups is frequently observed (Hansen and Moran, 2011).

Some insect species are more involved in symbiotic associations with bacteria than others. Among the insects, three taxonomic groups are regularly involved. These groups include Blattaria, Coleoptera, Homoptera, and Hymenoptera. Additionally, certain bacteria seem to be particularly adept at symbiotic interactions. Numerous arthropods carry representatives of the *Wolbachia* genus (Aikawa et al., 2022), which is closely linked to pathogenic *Rickettsia* (Shan et al., 2021) and is categorized in Proteobacteria’s subgroup. The subgroup contains symbiotic organisms closely related to significant human diseases, such as *Francisella tulariensis*, *Coxiella burnetii*, and several Enterobacteriaceae (Dang et al., 2017). Symbionts of mealybugs and the protist family Trypanosomatidae are members of the Proteobacteria β-subgroup (Boursaux-Eude and Gross, 2000). Cockroach mycetocyte symbionts (Blattaria) belong to the Flavobacterium–Bacteroides group (Guzman and Vilcinskas, 2020).

The maize weevil *Sitophilus zeamais*, for example, needs nutrients made by its endosymbiont *Sodalis pierantonius* to stay healthy. The symbionts’ innate immune system is generally activated by the weevils’ secretion of an antimicrobial peptide (AMP) in the microbiome, which prevents the weevils from generating a systemic antibacterial response against them (Wang et al., 2017; Maire et al., 2019; Trappeniers et al., 2019). When it comes to digesting plant tissues that are resistant to digestion, termites require more composite mutualism with lots of digestive-zone bacterial or protist species (Tokuda et al., 2018; Liu et al., 2019), and many of these microorganisms are termite-specific symbionts with a high degree of niche specialization (Bourguignon et al., 2018; Hervé et al., 2020). The microbiota of other various insects may be more varied and adaptable, as they do not rely on explicit critical symbionts (Coon et al., 2016; Scolari et al., 2019). The gut biota is critical for most insects’ digestion, fertility, fecundity, and immunity (Heys et al., 2018; Salcedo-Porras et al., 2020), as growing axenic insects can be deadly (Flury et al., 2019). Insects need to get several symbionts that successfully make good and functional microbiota.

Primary symbionts are more common in insects having particularly nutrient-deficient foods (obligate hematophagy or phytophagy). In contrast, secondary symbionts are more common in polyphagous and omnivorous insects, which obtain a diverse microbiota from their surroundings (Salcedo-Porras et al., 2020). While most primary symbionts are internal (endosymbionts), secondary symbionts are external. There may be an association between the symbiont acquisition or transmission and the nature of the interactions between insects and symbionts. It is usual for female germline transmission to occur vertically through the female germline as with primary mutualists, for example, those present in aphids and weevils (Caspi-Fluger et al., 2012; Douglas, 2015; Hassan et al., 2020). It is common for environmental microorganisms to be transmitted across internal organs, some of which can form secondary symbioses without specialized organs.

Additionally, insect growth affects the time during which microbiota are acquired horizontally. Except for vertically transmitted microorganisms, most insects hatch practically germ-free and obtain their microbiome by cannibalism, trophallaxis, coprophagy, or ingesting their contaminated eggshells (Taylor et al., 2014; Salcedo-Porras et al., 2020). Holometabolous insects pupate in a nearly axenic state, and adults re-acquire some of their gut microbiota from the environment (Powell et al., 2014; Hammer and Moran, 2019; Rolff et al., 2019) after emerging from the pupal stage. The microbiota of adults in some species may differ greatly from the microbiome of the immature stages or may acquire a similar gut microbiota from the conspecifics or environment (Johnston et al., 2019; Majumder et al., 2020; Suárez-Moo et al., 2020). On the contrary, microorganisms attained after egg hatching can be preserved in hemimetabolous insects for an extended period (Rodríguez-Ruano et al., 2018; Hammer and Moran, 2019). Finally, social insects, whether hemimetabolous or holometabolous, can get microbiota from each other repeatedly, choosing and keeping a specific microbiota (Onchuru et al., 2018; Tokuda et al., 2018; Liu et al., 2019).

## Acquisition Resistance Characteristics of Native Gut Bacteria

The increased predominance of naturally existing inhibitory gut bacteria could be a viable alternative to para-transgenic techniques for reducing pathogen burden in natural populations of insects’ vector. The configuration of the gut microbiome regulates vector capability by modulation of immunological reactions, competition for positions, or production of inhibitory compounds (Cirimotich et al., 2011; Boissière et al., 2012). The practical investigation of the gut microbiome to understand its contact with the parasite and host might lead to the development of innovative and more effective techniques to regulate vector-borne infections. As a result, future plant pest control efforts should consider this. Numerous microbial plant inflammations are conveyed *via* insect vectors, and the identification of these insects’ intestinal bacteria has been conducted to create techniques to prevent pathogen spread (Raddadi et al., 2011; Engel and Moran, 2013). An excellent example is a disease (Pierce) of grapes produced *via* pathogenic *Xylella fastidiosa*. *Alcaligenes xylosoxidans* was isolated as a bacterial symbiont since the sharpshooter (Cicadellidae) spreads *X. fastidiosa*.

These bacteria live in insect’s foregut, where they share space with *X. fastidiosa*, a bacterium that can be harmful to people. Because *A. xylosoxidans* are elated into the plants’ xylem by insects feeding on sap, it is more likely to spread to other insects. These properties make *A. xylosoxidans* a promising option for use as a bio-control mediator against *X. fastidiosa* establishment through modest position elimination or as a para-transgenic conveyer for providing anti-*Xylella* drugs among other applications (Miller, 2011).

## Impacts of Gut Microbiota on the Activity of Pesticides

The insect-associated microbial community is dynamic and responsive to various stressors (Zhang et al., 2022). The related microbiota, like the insect, is subject to natural selection pressure, and its composition can be influenced by variables such as dietary changes, food scarcity, and exposure to toxic substances (Adair and Douglas, 2017; Akami et al., 2022). The microbiota of hosts exposed to pesticides as a source of selection pressure may also assist the host in metabolizing these substances. It may act as a source of variation, resulting in the host’s reduced susceptibility to pesticides (Akami et al., 2019a,b). Pesticide-degrading bacteria are prevalent throughout nature and have been identified in a variety of insect orders, including Lepidoptera (Ramya et al., 2016b; de Almeida et al., 2017), Hemiptera (Kikuchi et al., 2012), Diptera (Cheng et al., 2017), and Coleoptera (Akami et al., 2019b). There has been evidence that resistant strains of bacteria from the gut of *Plutella xylostella* Linnaeus (Xia et al., 2018) and *Spodoptera frugiperda* (de Almeida et al., 2017) have the capacity to breakdown many pesticides (Gomes et al., 2020). The selection of *S. frugiperda* strains based on pesticide-guided selection led to selecting pesticide-degrading bacteria absent in the microbiota of vulnerable, unselected larvae (de Almeida et al., 2017).

The microbial population of an insect’s digestive tract comprises bacteria belonging to the phyla Firmicutes, Proteobacteria, Actinobacteria, and Bacterioidetes, all of which can impact the biology of hosts (Paniagua Voirol et al., 2018; Gomes et al., 2020). Research on *Spodoptera littoralis* (Boisduval) found that the microbial community was mostly made up of Firmicutes, especially *Enterococcus* (Chen et al., 2016; Higuita Palacio et al., 2021). Firmicutes are found in the digestive tracts of many lepidopteran larvae, even though the digestive tracts of larvae are suggested to be not very suitable for bacteria to live. This includes *Spodoptera litura* Fabricius (Thakur et al., 2016), *Manduca sexta* Linnaeus (Holt, 2013), *Helicoverpa armigera* Hubner (Yuan et al., 2021), and many other lepidopteran species (Mereghetti et al., 2017; Gomes et al., 2020). Bacteria belonging to the genus *Enterococcus* are known to create a variety of bacteriocins, which are potent antibacterial chemicals that can influence the composition of the gut microbial communities (Van Arnam et al., 2018). The highest relative amount of Enterococcus was reported in *S. frugiperda* populations from the laboratory and from natural fields (Gomes et al., 2020).

According to various studies, the intestinal bacteria of insects have been shown to break down multiple pesticides and interfere with the effectiveness of pesticides used to control them (Ramya et al., 2016a; Cheng et al., 2017; de Almeida et al., 2017). The Proteobacteria families (Enterobacteria, Pseudomonada, and Burkholderia) could break down acephate, chlorpyrifos, trichlorfon, lambda-cyhalothrin, and Spinosad, respectively (Kikuchi et al., 2012; de Almeida et al., 2017; Itoh et al., 2018b; Gomes et al., 2020). Similarly, Actinobacteria and Firmicutes bacteria have also been shown to have a role in the process of removing toxins from the environment (de Almeida, 2013; Ramya et al., 2016b). The resistant strain of *S. frugiperda* harbor gut bacteria *Enterococcus* (Firmicutes) that were able to break down the pesticides (chlorpyrifos, lambda-cyhalothrin, deltamethrin, spinosad, and lufenuron) (Gomes et al., 2020). According to previous studies, there are several gut symbionts of different insects (orders Coleoptera, Diptera, Hemiptera, and Lepidoptera) that detoxify the pesticides (classes Benzoylurea, Carbamate, Methoprene, Neonicotinoid, Organochloride, and Organophosphate) by the different species of genera *Acetobacter*, *Actinobacteria*, *Aeromonas*, *Arsenphonus*, *Burkholderia*, *Citrobacter*, *Clostridium*, *Enterococcus*, *Exiguobacterium*, *Lachnospiracease*, *Lactobacillus*, *Lysinibacillus*, *Microbacterium*, *Pseudomonas*, *Staphylococcus*, *Symbiotaphrina*, and *Wolbachia* (Table 2).

**TABLE 2:** List of insect gut microbiota involved in pesticide degradation.

Pesticides	Gut microbiota	Insect pests	References
Benzoylurea	*Enterococcus mundtii*	*Spodoptera frugiperda*	de Almeida et al., 2017
	*Microbacterium arborescens*		
	*Staphylococcus sciuri* subsp. *sciuri*		
Carbamate	*Pseudomonas melophthora*	*Rhagoletis pomonella*	Boush and Matsumura, 1967
Methoprene	*Clostridium* spp.	*Aedes* spp. and *Anopheles* spp.	Receveur et al., 2018; Giambò et al., 2021
	*Lysinibacillus* spp.		
	*Staphylococcus* spp.		
Neonicotinoid	*Acetobacter* spp.	*Drosophila melanogaster*	Chmiel et al., 2019
	*Lactobacillus* spp.		
	*Lactobacillus plantarum*		Giambò et al., 2021
	*Arsenphonus* spp.	*Nilaparvata lugens*	Pang et al., 2018
Organochloride	*Pseudomonas melophthora*	*Rhagoletis pomonella*	Boush and Matsumura, 1967
Organophosphate	*Microbacterium* sp.	*Anopheles stephensi*	Soltani et al., 2017
	*Exiguobacterium* sp.		
	*Aeromonas* spp.		
	*Pseudomonas* spp.		
	*Citrobacter* spp.	*Bactrocera dorsalis*	Cheng et al., 2017; Guo et al., 2017
	*Actinobacteria* spp.	*Bombyx mori*	Chen et al., 2020; Giambò et al., 2021
	*Staphylococcus* spp.		
	*Enterococcus* spp.		
	*Lachnospiracease* spp.		Li et al., 2020; Giambò et al., 2021
	*Burkholderia* spp.	*Cavelerius saccharivorus*	Kikuchi et al., 2012; Itoh et al., 2018a
	*Wolbachia spp.*	*Culex pipiens*	Berticat et al., 2002
	*Symbiotaphrina kochii*	*Lasioderma serricorne*	Shen and Dowd, 1991
	*Enterobacter aburiae*	*Plutella xylostella*	Ramya et al., 2016a
	*Bacillus cereus*		
	*Pantoea agglomerans*		
	*Enterococcus* spp.		Xia et al., 2018
	*Pseudomonas melophthora*	*Rhagoletis pomonella*	Boush and Matsumura, 1967
	*Pseudomonas* spp.	*Riptortus pedestris*	Kikuchi et al., 2012
	*Flavobacterium* spp.		
	*Burkholderia* spp.		Kikuchi et al., 2012; Itoh et al., 2018a
	*Burkholderia* spp.	*Cavelerius saccharivous*	Kikuchi et al., 2012
	*Delftia lacustris*	*Spodoptera frugiperda*	de Almeida et al., 2017
	*Enterococcus casseliflavus*		
	*Enterococcus mundtii*		
	*Leclercia adecarboxylata*		
	*Microbacterium paraoxydans*		
Oxadiazine	*Bacillis cereus*	*Plutella xylostella*	Ramya et al., 2016a
	*Gammaproteobacteria* spp.	*Blatella germanica*	Pietri et al., 2018
Pyrethroid	*Enterococcus casseliflavus*	*Spodoptera frugiperda*	de Almeida et al., 2017
	*Enterococcus mundtii*		
	*Pseudomonas stutzeri*		
	*Arthrobacter nicotinovorans*		
	*Enterococcus casseliflavus*		
Spinosyn	*Enterococcus casseliflavus*		
	*Enterococcus mundtii*		
	*Pseudomonas psychrotolerans*		

**FIGURE 3:**
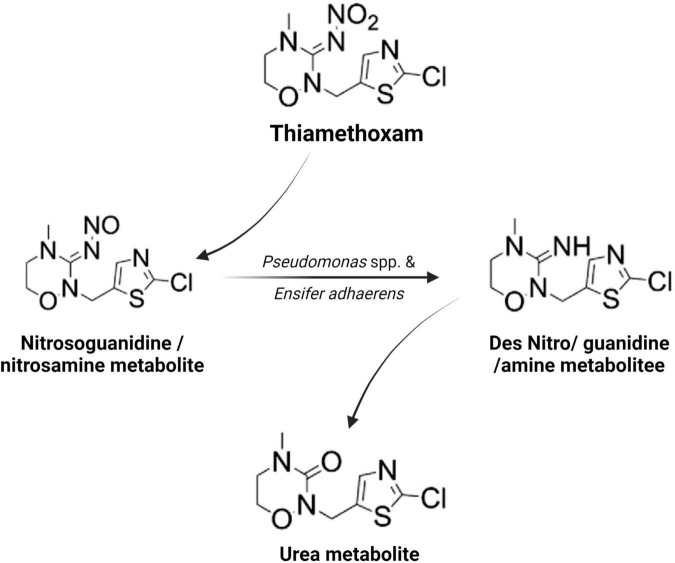
Metabolic routes for bacterial degradation of the insecticide thiamethoxam (Hussain et al., 2016).

**FIGURE 4:**
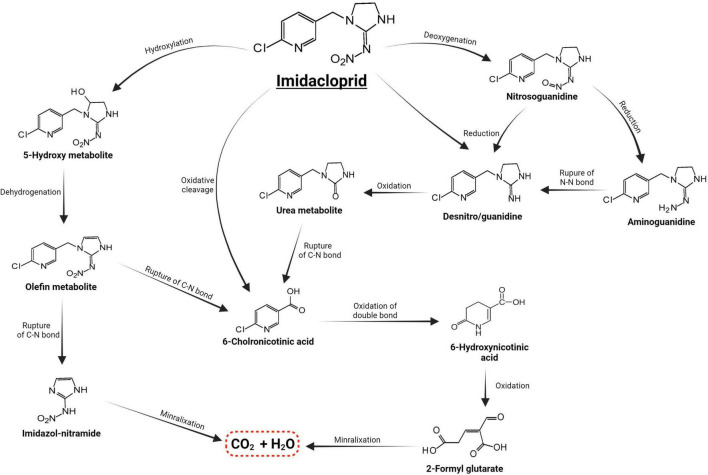
Metabolic routes for bacterial degradation of the insecticide imidacloprid (Pang et al., 2020a).

Microorganisms’ ability to utilize pesticides as a carbon source is contingent upon the coding of the biochemical systems required to cope with these substrates (Lourthuraj et al., 2022). Temperature and pH, nutrition availability, chemical concentration, and the size of the bacterial population all influence pesticide metabolization (Russell et al., 2011; Gomes et al., 2020). The pesticides’ chemical composition and complexity play a role in how quickly and effectively bacteria use them as a food source (Hubbard et al., 2014). Microorganisms use a wide range of metabolic pathways to break down and change xenobiotics when they grow rapidly (Itoh et al., 2018b; Bhatt et al., 2019, 2021; Gangola et al., 2022)—for example, *Pseudomonas* spp. and *Ensifer adhaerens* metabolized the thiamethoxam pesticide. The principal metabolic pathway involves the transition of its N-nitroimino group (= N-NO_2_) to N-nitrosimine/nitrosoguanidine (= N-NO, THX-II) and urea (= O; THX-III) metabolites (Hussain et al., 2016), which is shown in Figure 3. Another example is the symbionts species of genera *Arsenophonus* (Pang et al., 2018) and *Pseudomonas* (Pang et al., 2020b); *Ensifer* spp., *Stenotrophomonas* spp., *Variovorax* spp. (Hussain et al., 2016) have been reported to degrade imidacloprid. The mechanisms and associated metabolic pathways are shown in Figure 4, which indicates that nitro-reduction and oxidation are two of the main ways that bacteria break down imidacloprid (Lu et al., 2016; Fusetto et al., 2017). The gut microbiota produces enzymes that detoxify pesticides like pyrethroids, carbamates, diamides, and organochlorines, which have been identified (Russell et al., 2011; Khalid et al., 2016; Gomes et al., 2020; Lin et al., 2022).

Moreover, Plant secondary components, such as terpenes, alkaloids, glycosides, and phenolic compounds, are degraded by Proteobacteria in the presence of insects (Mereghetti et al., 2017; Gomes et al., 2020). Proteobacteria have the most diverse morphology and adaptability of all bacterial phylum, which offers them an advantage in various ecological niches (Shin et al., 2015). Proteobacteria may thus act as a source of available variety and a tool for host adaptation in nature when they interact with other organisms (Bradley and Pollard, 2017; Degli Esposti and Martinez Romero, 2017; Hauffe and Barelli, 2019).

The gut microbiota has been linked to promoting the insecticidal action of *Bacillus thuringiensis*, the frequently used biological pesticide for herbivore pest management in agriculture (Mason et al., 2011; Eski et al., 2018). According to a study, when the gut microbial population was removed from gypsy moth larvae, the *B. thuringiensis* pesticide no longer worked as intended, whereas when some microbiota of the gut microbiome was added back in, the *B. thuringiensis*-facilitated mortality was reestablished (Polenogova et al., 2021). Several insect species have similar mechanisms for degrading imidacloprid (Thurman et al., 2013). Additionally, the Cyp6g1 gene discovered in *Drosophila* is critical for imidacloprid breakdown in animals, regulating and promoting the generation of metabolites in the oxidation pathway (Fusetto et al., 2017). These findings indicate the importance of considering the gut microbiome of insects in the development of novel pest control methods.

## Symbiont-Mediated Pesticide Resistance

The rapid emergence of pesticide resistance in a wide range of organisms is a cause for concern, and it merits additional studies. Several mechanisms of pesticide resistance are ascribed to the physiology at the host level (Tabashnik and Carrière, 2010; van den Bosch and Welte, 2017), but few researchers have argued in recent years that some pesticides’ resistance might be ascribed by symbiont detoxification. Detoxifying enzymes targeting harmful allelochemicals and pesticides have been found in fungal symbionts isolated from insects (Tabashnik and Carrière, 2010; Naik et al., 2018). Evidence showing that changes in mutualist-level physiology can cause pesticide resistance is very limited—for example, the *Burkholderia* mutualist in midgut crypts acquired *via* the environment in each generation instead of the “traditional” mode of vertical maternal spread, as is the case with humans (Garcia, 2015). The pesticide fenitrothion, a common organophosphorus chemical in agriculture, can be degraded by the symbiotic *Burkholderia* (Kikuchi et al., 2012).

Furthermore, the *R. pedestris* pest bug easily forms symbiotic relationships through fenitrothion, degrading *Burkholderia* mutualists, and have significantly more persistence rates on fenitrothion-treated plants than insects by non-demeaning *Burkholderia* mutualists (Kikuchi et al., 2012). Spraying of fenitrothion to the field enabled more bacteria to degrade fenitrothion in soil, which is thought to impact the dynamics of symbiotic-degrading *Burkholderia* spread *via* soil to stinkbugs (Tago et al., 2015). These discoveries imply that pesticide resistance may mature in the absence of pest insects in a field and then spread rapidly within sole insect pest generation (Kikuchi et al., 2012). In addition, *Burkholderia* symbionts of the established *R. pedestris* model give an excellent chance for research on microbial symbiotic aspects at the molecular level since they are cultivable and genetically manipulable (Kim and Lee, 2015). These studies help develop an ecological pesticide that uses gut symbionts to control insects. Such studies could be useful to learn about pesticide resistance mechanisms that have not been found yet.

## Molecular Mechanisms by Which Enzymes Mediate Pesticide Detoxification

Detoxification enzymes occur naturally in various biological processes, functioning on the target sites to neutralize various toxins prevalent in the insect body (Lin et al., 2015; Bhandari et al., 2021; Siddiqui et al., 2022). According to some previous studies, the biochemical characterization of insect resistance to pesticides is connected to pesticide sensitivity at the target site and pesticide detoxification by metabolic enzymes (acetylcholinesterase, carboxylesterase, glutathione S-transferase, and cytochrome P450) (Wu et al., 2014; Ismail, 2020; Yang et al., 2021; Siddiqui et al., 2022). These enzymes are crucial in detoxifying xenobiotics (Hu et al., 2014), where their hosts can utilize these enzymes as biological indicators during pesticide detoxification (Khan et al., 2021)—for instance, Zhang et al. (2016) reported that detoxification enzymes (cytochrome P450 genes) were detected during detoxification of fipronil in the red imported fire ants (*Solenopsis invicta* Buren), where up to 36.4-fold rise in resistance was recorded following exposure of the ants to fipronil. Another related study has also linked cytochrome P450 enzymes with fluralaner detoxification in *S. invicta* (Xiong et al., 2020).

Additionally, these enzymes may raise the responder gene’s copy number, mRNA levels, and coding sequence diversity by introducing point mutations (Pang et al., 2020a). These enzymes are involved in various processes, including biosynthesis and the metabolism of invading species, among others—for instance, P450 CYP6ER1 in *Nilaparvata lugens* and CYP6CM1 in *Bemisia tabaci* were used to characterize and assess imidacloprid metabolism. These findings revealed that amino acid changes in the binding site enhanced imidacloprid metabolism (Bao et al., 2016; Pang et al., 2016; Hamada et al., 2019). Puinean et al. (2010) discovered that the CYP6Y3 gene in *Myzus persicae* could confer resistance to neonicotinoids. Moreover, CYP353D1v2 was found to be overexpressed in several imidacloprid-resistant *Laodelphax striatellus* strains, and silencing this gene greatly reduced resistance (Elzaki et al., 2017). The effective suppression of CYP6CY14 transcription by RNAi in the overexpressed P450 gene of the CYP3 clade greatly improved the vulnerability of pesticide-resistant cotton aphids to thiamethoxam (Wu Y. et al., 2018).

In order to detoxify xenobiotics in the gut lumen, insects can employ various techniques. They can do so by creating an acidic environment and supplying a complex of enzymes (monooxygenases and esterases) that can cleave or alter the xenobiotic in preparation for excretion (de Almeida et al., 2017). It has been confirmed that microbial enzymatic activity in the gut lumen contributes to the breakdown of pesticides consumed by the host. The hydrolysis of these compounds provides resources for the microbiota to thrive (Mohammadi et al., 2021). The diversity and differences in prokaryote- and eukaryote-produced enzymes suggest that microbial enzymes could play a significant role in pesticide metabolization in contaminated insects (Russell et al., 2011; de Almeida et al., 2017).

## Pesticide Degradation by Symbionts in Invasive Species

Multiple resistance mechanisms have been functionally recognized as conveying pesticide resistance in several invasive insects, including penetration resistance *via* cuticle thickening or remodeling, metabolic resistance *via* the amplified activity of detoxification enzymes (e.g., esterases and cytochrome P450 monooxygenases), and knockdown resistance *via* kdr transmutations (Khan et al., 2021; Rigby et al., 2021). There are also possible behavioral and physiological resistance mechanisms. These include point mutations that make esterases more active, GST, target place insensitivity, reformed AChE, GABA receptor insensitivity, and transformed nAChRs (Dang et al., 2017). The diamondback moth, *Plutella xylostella*, is an example of invasive species that act as a significant universal pest of various crops (Nakaishi et al., 2018). *P. xylostella* also generates an enzyme that avoids the generation of dietetic isothiocyanates that act as plant defense compounds emitted by the host plant and regulate feeding behavior of diamondback moths female (Hussain et al., 2019, 2020). Furthermore, *P. xylostella* has been discovered to be resistant to a wide range of chemical pesticides. Only three other pest species have established resistance to *Bacillus thuringiensis*-based pest control technologies, which is one of them (Furlong et al., 2013). The quick evolution of extremely resistant phenotypes in *P. xylostella* is partly ascribed to insect pests, including altered carbamate and organophosphate target locations, parathion metabolism by GST, and pyrethroid detoxification by P-450 monooxygenases (Ramya et al., 2016a). The indoxacarb-degrading microbiota (*B. cereus* bacteria) identified in the digestive tract of *P. xylostella* was found to degrade the pesticide by converting it into food (van den Bosch and Welte, 2017). Another pesticide, acephate, was quickly degraded by gut bacteria obtained from diamondback moth intestines. Together with earlier research on the gut microbiota of stinkbugs showing pesticide resistance (Kikuchi et al., 2012), these findings suggest that the gut biota might have a greater part in pesticide resistance than formerly assumed.

## Role of Gut Microbiota in Tolerance and Resistance

Insect’s digestive systems are equipped with a multilevel defensive system, likely a primary driver in structuring gut microbiome communities. Different aspects of such a defensive system provide the host’s ability to tolerate and reject harmful bacteria in the gut through various processes. While tolerance reduces the detrimental effects of a bacterial burden on the host’s health, resistance reduces the bacterial burden so as not to harm the host (Moreno-García et al., 2014). Most immunological research have concentrated on resistance mechanisms, and there is little knowledge about the processes that mediate tolerance. However, host–microorganism associations in the insect gut are frequently commensalism or mutualism.

Compared to insects with sparsely populated digestive tracts, those with vast bacterial communities are more likely to be tolerant and less likely to be resistant to bacteria in their guts. As a result, the gut immunity mechanisms of diverse insects may be tailored to the definite desires of their hosts. As mentioned previously, the midguts of most insects produce a peritrophic medium composed of a network of chitin microfibrils implanted in a protein–carbohydrate medium (Muthukrishnan et al., 2012). The peritrophic medium is semi-permeable, allowing nutrients, digestive enzymes, and defense chemicals to flow while protecting the epithelial cell layer from a direct microbe or toxin exposure. The cuticle layer bordering the epithelial cell layer in the foregut and hindgut may have comparable protective roles.

These physiological barriers among the lumen and epithelium are decent instances of tolerance mechanisms since they minimize the influence of bacteria on the host rather than reduce the bacterial load in the gut. Certain parts of the insect gut can have a low or high pH or contain enzymes that target bacterial cell wall components, such as peptidoglycan or lysozymes (PGN) hydrolases (Liu et al., 2014; Moreno-García et al., 2014). Such systems can cause deliberate resistance by reducing the number of bacterial communities in specific parts of the gut, but they may be useful in bacterial cell breakdown to enhance nutrition.

Bacterial endosymbionts have been extensively examined in the context of biological invasions to detect or quantify their impact in increasing the invasion process of imported species (Klock et al., 2015; Taerum et al., 2016; Cheng et al., 2019). There is still a lack of understanding of the mechanisms that drive the responses of native species to invasive species’ selective pressures. A deeper knowledge of the structure and function of bacterial mutualists, on the other hand, may disclose possible mechanisms for inhabitant hosts to adapt to exotic species, as variations in bacterial mutualists have been revealed to correlate with variations in food sources in both invertebrates and vertebrates (Rokhsefat et al., 2016; Shapira, 2016). Furthermore, identifying and describing the bacterial mutualists of inhabitant species may supply vital hints about handling exotic species in the future.

Plant defenses and pesticides can potentially interact with and supplement host immune systems (Mason, 2020). Secondary metabolites play a critical role in protecting plants from arthropod herbivores. Secondary chemicals play an important role in insect resistance and vulnerability (Erb and Kliebenstein, 2020). Plant secondary metabolites with antinutritive, deterring, antibacterial, and poisonous properties frequently affect the growth and productivity of phytophagous insects feeding on various host plants (Puri et al., 2022). In nature, the level of plant-defensive compounds varies by species and is determined by the plant’s genotype, growing circumstances, and phenology. Plant allelochemicals impose a very strong selection pressure on herbivorous insects and the microbiota in their guts, which is particularly important for their survival (Douglas, 2015; Chen et al., 2022), for example, a study discovered that symbionts such as *Phenylobacterium*, *Ochrobactrum*, *Erwinia*, *Amycolatopsis*, and *Sediminibacterium* spp. may play critical roles in the metabolism of tea saponins, according to the findings. Two of them, *Acinetobacter calcoaceticus* and *Acinetobacter oleivorans*, were very important in the degradation of tea saponins (Zhang et al., 2020b).

The gut microorganisms’ digesting abilities can also assist in the removal or inactivation of toxic compounds in food (Schmidt and Engel, 2021). Detoxification symbioses have been observed in a wide range of hosts, even though certain insects have these functions encoded in their genomes (Itoh et al., 2018b). They are particularly important for herbivorous insects since plants produce a diverse spectrum of phytotoxins that are toxic to them (Itoh et al., 2018b). Adaptation to the highly toxic terpenoids present in the bark of pine trees has been achieved by cooperation between the mountain pine beetle (*Dendroctonus ponerosae*) and the pine weevil (*Hylbius abietis*) and their gut microbiota. Gammaproteobacteria, in particular, play an important role in the degradation of diterpenes (Berasategui et al., 2017; Schmidt and Engel, 2021).

An example of a social insect belonging to the genera *Apis* and *Bombus* harbors gut microbiota that plays important roles in their health, with a possible impact on pathogen protection and nutrient acquisition (Engel et al., 2016; Zheng et al., 2016). Glycolysis pathways and phosphotransferase systems have been found in the genomes of *Gilliamella apicola*, indicating that this bacterium functions as a saccharolytic fermenter that aids in the digestion of the host’s carbohydrate-rich meal (Tilottama et al., 2021). The pollen grain of *G. apicola* was subjected to a metagenomic investigation, and the results revealed the presence of genes encoding pectin-degrading enzymes. These enzymes play a vital role in breaking down the stiff polysaccharide walls of pollen grains and release constituent monosaccharides (Zheng et al., 2016).

The coffee borer beetle (*Hypothenemus hampei*) engages in a detoxifying symbiosis to facilitate nutritional adaption to coffee beans, which contain high quantities of the poisonous alkaloid caffeine (Mejía-Alvarado et al., 2021). It was discovered that the beetle’s gut microbiota is dominated by *Pseudomonas* species, which are seen in beetles from several coffee-producing countries (Ceja-Navarro et al., 2015). Beetle pseudomonad spores were able to develop on caffeine alone, and they were able to restore the breakdown of caffeine in beetles that had been previously treated with antibiotics (Schmidt and Engel, 2021). In addition to promoting nutritional adaptability, it has been observed that several pest species carry gut symbionts that are capable of degrading pesticides (de Almeida et al., 2017; Itoh et al., 2018b). The *Burkholderia* gut symbiont, *R. pedestris*, may degrade the pesticide fenitrothion and increase the survival of *R. pedestris* in soil infected with the pesticide (Itoh et al., 2018a). The wasp *Nasonia vitripennis*, for example, was found to have a greater survival rate in the presence of its gut microbiota in the exposure of atrazine (Wang et al., 2020). This research highlights the gut microbiota’s ability to boost the adaptive capabilities of its insect host, which has crucial implications for pest and pollinator insect control.

**FIGURE 5:**
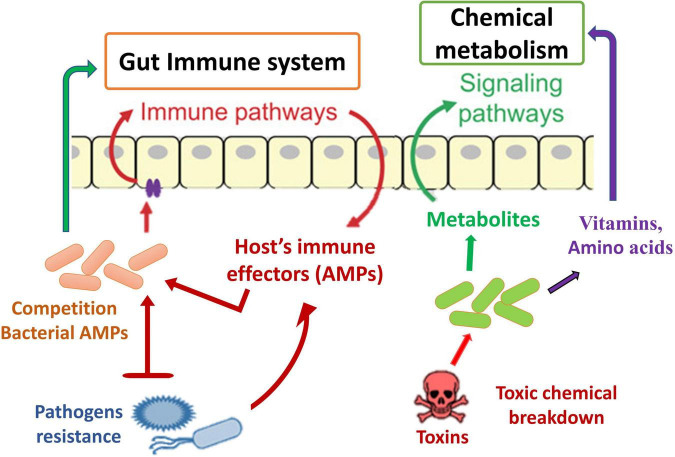
Schematic diagram of the role of gut microbiota in immune and resistance mechanism.

Another defense mechanism is the inherent immune system of insect species, which comprises numerous immunological responses (Chambers et al., 2012; Engel and Moran, 2013) and summarizes the general principles of innate immunity in insects. A key inducible response permitting resident immunity at the gut epithelial cell layer has been identified, mostly through experiments with *D. melanogaster*. These are the creation of amino acids (AMPs) (Figure 5) and the combination of reactive oxygen species (ROS). The generated reactions may altogether be considered traditional resistance mechanisms; nevertheless, they contain undesirable response circles and modulatory mechanisms, conveying host tolerance toward the commensal gut microbiota. The Toll and IMD signaling channels are two of the most important signaling mechanisms causing AMP synthesis in *D. melanogaster*’s systemic immune response (Hanson and Lemaitre, 2020). The reaction in the gut is distinct in that only the IMD pathway is activated, resulting in the induction of resident AMP reactions in response to pathogen stimulation (Nehme et al., 2007; Lee et al., 2013). Initiation happens when different types of bacterial PGN bind to receptors on the outside or inside of the body’s epithelium that belong to the peptidoglycan recognition protein (PGRP) family (Engel and Moran, 2013). Signaling downstream *via* the IMD pathway activates the transcriptome factor Relish, which stimulates the production of multiple AMPs and other immunity-associated genes (Figure 5). Introduction to pathogens also results in ROS formation in the gut of *D. melanogaster* through the membrane-related dual oxidase (DUOX) system (Alaraby et al., 2018). The PGN-independent and PGN-dependent signaling pathways are involved in this process (Charroux and Royet, 2012). In addition to the bacteria, the host’s epithelial cells are also subjected to oxidative stress when ROS are produced.

Immune catalases are activated in *D. melanogaster* to remove excess ROS (Buchon et al., 2014). Tolerance is improved due to this catalase synthesis, which reduces the immune response-induced self-harm (Simpson et al., 2015). How these enzymes protect the host cells without affecting the pathogens’ ability to produce ROS remains a mystery. One possible reason is that the catalase activity is constrained to a specific region of the epithelial surface—for instance, the vicinity of the epithelial surface. Immunological reactions to the related gut microbiome community have been confirmed in *D. melanogaster* by employing the DUOX system activity and IMD pathway at varying degrees. In the IMD pathway, the homeobox transcript factor caudal binds to the promoter regions of AMP genes in the gut and stops them from being made. The gut flora alters, and the epithelial cell layer breaks down in caudal-defective flies because of a constant generation of AMP (Ryu et al., 2008; Broderick and Lemaitre, 2012). As a result, it appears that caudal avoids over-encouragement of the immunity system by mutualistic gut biota. Additional immunological regulatory operations in *D. melanogaster* are regulated by amidases produced by the midgut cells of the epithelium and cleave pro-inflammatory PGN into passive systems (Bischoff et al., 2006; Zaidman-Rémy et al., 2006; Engel and Moran, 2013).

There are many ways in which obligatory insect-associated bacteria contribute to their host insect’s overall health and well-being; however, the primary contribution of these bacteria is connected only to their ability to provide nutrients. Secondary bacterial symbionts boost the host’s immunological response to entomophagy (Vorburger et al., 2010) and entomopathogens (Jaenike et al., 2010), impact host plant selection (Frago et al., 2012), defend against heat stress (Pons et al., 2022), and aid in the detoxification of compounds produced for herbivore defense (Hammer and Bowers, 2015). Microbes also plays a role in detoxifying xenobiotics by catabolizing organic compounds used in applied pest management, as demonstrated by degradation (Pietri et al., 2018).

## Conclusion and Future Perspectives

Microbes are known to degrade a wide variety of allelochemicals and pesticides, providing numerous opportunities for insects to develop detoxifying symbiotic relationships. The gut microbiota plays various roles in the host’s physiology, including immunological modulation and toxin degradations. Arguably, the microbiota evolves more rapidly than their host insects, resulting in rapid pest adaptation to pesticides through the employment of mutualistic microbes. Additionally, insects can swiftly obtain novel metabolic activities and colonize new ecological niches through symbiotic interactions with microbiota that previously have fully developed well-tuned metabolic pathways. As results of the ever-dynamic climatic conditions and human populations, it is imperative that additional/novel insect pest management strategies are implemented to synergize the existing ones. Exploring symbiotic microorganisms as a means of managing their associated hosts could be one way to meet this need. Currently, sterile insect technology, introduction of natural enemies such as parasitoids or predators, application of entomopathogenic fungi or bacteria, etc., are some of the most commonly used integrated pest management techniques. Additional research into (detoxifying) symbiosis may result in environmentally acceptable and long-term ways of controlling large pest insect populations. Insect pest status, for example, may be heavily influenced by microbiota genotype, allowing for the identification and selection of genotypes most suited for addressing specific pest management priorities, ideally through low-tech means. In the same vein, detoxifying microbiota that can be isolated could be used in bioremediation or to treat pesticide poisoning. To better understand detoxifying microbiota in agriculturally significant pest insects, we provided comprehensive information regarding the role of gut microbiota in the detoxification of pesticides. Further investigation may be helpful to produce an effective integrated pest management program.

## Author Contributions

JS, MK, BB, and MH wrote the initial draft. YX financially supported and supervised the manuscript. JS and YX conceptualized and developed the document. MQ and MTR provided critical feedback and reviewed the manuscript. MAR, SA, and MS revised the manuscript. All authors have read and agreed to the final version of the manuscript.

## Conflict of Interest

The authors declare that the research was conducted in the absence of any commercial or financial relationships that could be construed as a potential conflict of interest.

## Publisher’s Note

All claims expressed in this article are solely those of the authors and do not necessarily represent those of their affiliated organizations, or those of the publisher, the editors and the reviewers. Any product that may be evaluated in this article, or claim that may be made by its manufacturer, is not guaranteed or endorsed by the publisher.
